# GDF15 and Growth Control

**DOI:** 10.3389/fphys.2018.01712

**Published:** 2018-11-27

**Authors:** Paul J. Emmerson, Kevin L. Duffin, Sudhakar Chintharlapalli, Xinle Wu

**Affiliations:** ^1^Lilly Research Laboratories, Eli Lilly and Company, Indianapolis, IN, United States; ^2^Lilly China Innovation and Partnerships, Shanghai, China

**Keywords:** GDF15, TGFβ, GFRAL, obesity, metabolic, cancer, cardiovascular

## Abstract

Growth/differentiation factor-15 (GDF-15) is a distant member of the transforming growth factor β (TGF-β) superfamily and is widely expressed in multiple mammalian tissues. Its expression is highly regulated and is often induced in response to conditions associated with cellular stress. GDF15 serum levels have a strong association with many diseases, including inflammation, cancer, cardiovascular diseases, and obesity, and potentially serve as reliable predictor of disease progression. A functional role for GDF15 has been suggested in cancer, cardiovascular disease, kidney disease and metabolic disease. However, the knowledge of its pathophysiological function at the molecular level is still limited and requires more investigation. Recent identification of the endogenous receptor for GDF15 may provide additional insight in to its’ molecular mechanisms and relationship to disease states.

## Introduction

Growth/differentiation factor-15 (GDF-15), also known as MIC-1, NAG-1, PLAB, or PTGFB, was discovered as a remote member of the transforming growth factor β (TGF-β) superfamily more than 20 years ago (Bootcov et al., 1997; Hromas et al., 1997; Lawton et al., 1997). During an effort to identify genes expressed in association with macrophage activation, a subtraction cDNA library comparing phorbol 12 myristate 13-acetate (PMA) treated and untreated human monocytoid cell line U937 was screened. Macrophage inhibitory cytokine 1 (MIC-1) was identified as a novel gene that encodes a protein containing structural characteristics similar to TGF-β (Bootcov et al., 1997). Expression of MIC-1 mRNA in macrophages can be up-regulated by a variety of stimuli, such as interleukin 1β (IL1β), tumor necrosis factor α (TNF-α), interleukin 2 (IL2), and macrophage colony-stimulating factor (M-CSF) (Bootcov et al., 1997). A similar approach was used to identify genes regulated by cyclooxygenase (COX) inhibitors. A cDNA library was built from the human colorectal cell line, HCT-116, treated with nonsteroidal anti-inflammatory drugs (NSAIDs). NSAID activated gene (NAG-1) was identified from this library and was found to have a sequence identical to MIC-1 (Baek et al., 2001). In another two separate efforts to identify novel TGF-β superfamily members and bone morphogenetic proteins (BMPs) enriched in placenta, the same gene was also cloned and termed as PLAB (placental bone morphogenetic protein) and Placental Transforming Growth Factor Beta (PTGFB) (Hromas et al., 1997; Lawton et al., 1997). Bottner et al. (1999) identified GDF15 by screening the human placenta-derived EST database with the “cystine knot” motif, a common signature of the members of the TGF-β superfamily.

The human GDF15 gene is located on chromosome 19p13.1–13.2 as determined by *in situ* hybridization. The human, rat and mouse GDF15 genes are all composed of two exons, and contain one single intron that interrupts the coding sequences in the pre-pro-domain of the corresponding proteins (Bottner et al., 1999). The mature protein sequence contains the seven conserved cysteine residues that form the cysteine knot. Similar to the other TGF-β superfamily members, full length GDF15 contains a pre-pro-domain and the mature protein is generated after cleavage at a furin-like site. The sequence conservation of GDF15 between species is relatively low, and the respective orthologs show 70% similarity, which is the lowest within the TGF-β superfamily (Bottner et al., 1999).

Growth/differentiation factor-15 (GDF-15) is associated with multiple diseases and its phenotypic functions are summarized in Table 1.

**Table 1:** List of Growth differentiation factor-15 (GDF-15) target tissues, organ and functions, mechanism.

Target Tissue/Organ	Function/Mechanism
Tumor	Both pro and anti-tumorigenic roles
Cardiovascular System	Inhibit myocardial hypertrophy, pro and anti-atherosclerosis
Kidney	Renal protective, anti-fibrosis
Brain stem	Suppress appetite through activation of GFRAL receptor in the area postrema


## GDF15 and Stress Response

Growth/differentiation factor-15 (GDF-15) mRNA has a wide tissue distribution pattern. It is highly expressed in the placenta and prostate but can also be detected in the heart, pancreas, liver, kidney, intestine, lung and colon (Yokoyama-Kobayashi et al., 1997; Bottner et al., 1999; Tan et al., 2000; Koopmann et al., 2004). GDF15 protein is produced as a 35 kDa full length form. After the N terminus is cleaved, the mature protein is secreted as a 25 kDa disulfide linked dimer and its circulation level can be easily quantified (Bootcov et al., 1997).

Studies have shown that GDF15 is a stress-induced cytokine released in response to tissue injury. For example, Hsiao et al. (2000) reported that Gdf15 expression in the liver increased significantly and rapidly following administration of chemicals known to injure hepatocytes such as CCl4. Besides chemical injury, they also determined that Gdf15 mRNA expression was induced following partial hepatectomy. Gdf15 expression can be induced after not only direct assault on hepatocytes, but also following the injury to bile duct epithelial cells. Chemical bile duct injury induced by methylenedianiline (DAPM) led to increased Gdf15 mRNA levels. Interestingly, *in situ* hybridization confirmed Gdf15 mRNA is induced in the hepatocytes, whereas no Gdf15 mRNA was detected in the bile duct regions, the primary injury site induced by DAPM. However, despite the dramatic induction pattern following liver injury, Gdf15 does not seem to play a significant role in liver injury or regeneration. Gdf15 null mice regenerated their liver mass at the same rate as the wild-type mice after partial hepatectomy.

Zimmers et al. (2005) investigated the regulation of Gdf15 in murine models of kidney and lung injury. Following CCl4 injection, increased GDF-15 protein expression in kidney and lung was detected by western blotting. In the five/sixths nephrectomy and kidney and ischemia reperfusion models, Gdf15 mRNA was increased as determined by northern blotting analysis. They also developed two models of lung injury, bleomycin administration in adult mice and prolonged hyperoxic exposure in neonates. In both cases, Gdf15 mRNA was markedly induced.

Gdf15 expression also increased rapidly in response to cardiovascular injury. For example, in a mouse model of myocyte enhance factor 2C (MEF2C) overexpression in the heart, which causes dilated cardiomyopathy, Gdf15 mRNA was significantly induced in the cardiomyopathic heart of adult mice. Increased Gdf15 protein expression was also confirmed by Western Blot (Xu et al., 2006). In an ischemia model, permanent coronary artery ligation was performed on mice and a rapid induction of Gdf15 mRNA was detected in the ischemic area. Coronary reperfusion after temporary ischemia also resulted in an increase of Gdf15 mRNA in the area at risk (Kempf et al., 2006).

## GDF15 as a Biomarker

Because GDF15 protein concentration can be easily determined in the circulation, many studies have identified it as an important plasma biomarker that correlates with various diseases. GDF15 is associated with cardiovascular diseases such as heart failure, coronary artery disease, and atrial fibrillation. Increased circulating GDF15 levels are also found in diabetes, cancer, cognitive impairment and cachexia patients (Johnen et al., 2007; Adela and Banerjee, 2015; Tsai et al., 2016; Wollert et al., 2017). In a case-control study of initially healthy women, baseline concentrations of GDF15 were established in 257 women who had a myocardial infarction, stroke, or died from a cardiovascular event during a 4 year follow-up period and in 257 age and smoking status matched women who did not report cardiovascular disease. Women who developed cardiovascular events had higher baseline GDF15 concentrations. Concentrations above the 90th percentile were associated with a 2.7-fold increase in risk. This effect was independent of traditional cardiovascular risk factors such as C-reactive protein (Brown et al., 2002). Another study examined GDF15 levels in 14577 patients with stable coronary heart disease and found GDF15 levels to be associated with cardiovascular death, sudden death, heart failure death, cancer death, and hospitalization for heart failure (Hagstrom et al., 2017). The association between GDF15 and cardiovascular outcomes was also studies in the Framingham Heart Study (3428 participants, mean age: 59 years; 53% women). GDF15 was associated with all-cause mortality, incident heart failure, and major cardiovascular events (Wang et al., 2012). The association of GDF15 with all-cause mortality was further explored in 1391 Rancho Bernardo Study participants, mean age 70 years, with no history of cardiovascular disease and followed for a mean of 11 years. GDF15 was found to be a strong predictor of all-cause and non-cardiovascular mortality (Daniels et al., 2011).

Brown et al. (2003) measured serum level of GDF15 from 260 healthy blood donors and 193 patients with adenomatous polyps or colorectal carcinoma. There was a progressive increase in GDF15 levels in those with adenomatous polyps or colorectal carcinoma. Serum GDF15 level also correlated with the Tumor-Node-Metastasis stage (Brown et al., 2003). In a retrospective study, GDF15 serum level was determined in 70 samples from prostate cancer patients and normal and biopsy-negative individuals. GDF15 concentration was elevated in prostate cancer patients and correlated with the progression of cancer (Li et al., 2015).

## GDF15 and Cancer

As discussed in the previous section, elevated GDF15 level in serum is associated with many cancers. Several studies showed that higher expression of GDF15 mRNA and protein in cancer biopsy samples (Welsh et al., 2001, 2003). However, functional studies of the roles played by GDF15 in cancer is limited and controversial. Some data suggest that GDF15 has tumor suppressor activity, while other results suggests the opposite.

### Tumor Suppressor Activity

To assess the impact of GDF15 on tumor growth, MCF-7 cells overexpressing GDF15 were injected into the mammary fat pad of nude mice in an orthotopic tumor model. The overexpression of NAG-1 was found to reduce the size of the orthotopic tumor by approximately 50% (Martinez et al., 2006). In another study, PC-3 cells transfected either with empty vector or GDF15 over-expression constructs were injected subcutaneously into the abdomen of athymic nude rats. Tumors generated from the empty vector-transfected control cells grew rapidly throughout the time course while GDF15-transfected cells grew at a much-reduced rate (Lambert et al., 2006). A GDF15 overexpressing clone of the LN-Z308 cell line was injected subcutaneously into the flanks of five Swiss nude mice, and the tumorigenic activity was completely abolished (Albertoni et al., 2002). Tumor suppressor activity of GDF15 was also tested in a transgenic mouse model. Gdf15 transgenic mice were treated with the colorectal carcinogen azoxymethane. In comparison to the non-transgenic littermates, transgenic mice developed 50% fewer aberrant crypt foci and no tumors. After crossing with ApcMin mice, a mouse model for intestinal tumorigenesis, double heterozygotes had a significantly reduced polyp load (60%) compared with nontransgenic ApcMin littermates (Baek et al., 2006). GDF15 transgenic mice were also crossed with the Transgenic Adenocarcinoma of Mouse Prostate (TRAMP) transgenic model of spontaneous prostate cancer. Overexpression of GDF15 prolonged survival and restrained cancer growth in TRAMP mice (Husaini et al., 2012). Conversely, loss of GDF15 led to stimulation of tumor growth. Inhibition of intestinal polyp formation by the COX inhibitor sulindac was lost in GDF15 deficient ApcMin mice (Zimmers et al., 2010).

### Pro-tumorigenic Activity

Boyle et al. (2009) investigated the effect of GDF15 on the growth of tumors in a mouse xenograft model. They transfected D04, A2058, and C32 metastatic melanoma cells with either a control shRNA or a specific shRNA for Gdf15. Cells were injected subcutaneously into nude mice. The cells treated with control shRNA rapidly developed into visible tumors, while knocking down of Gdf15 resulted in minimal tumor formation. To evaluate the role of GDF15 in the prostate cancer, PC-3 cells overexpressing GDF15 were generated and injected into the prostate gland of athymic nude mice. Compared to the animals injected with PC-3 cells transfected with the empty vector, there was no significant difference in the tumor weight.

Several studies have shown that GDF15 plays an important role in suppressing the tumor progression. At the same time, literature shows overexpression of GDF15 in tumors, which contradicts the tumor suppressor activity of GDF15. This discrepancy may be due to the tumor suppressor activity of GDF15 in early stages of tumor development and progression and later becoming a tumor promoter as the tumor progresses into a malignant tumor. Other members of TGFβ superfamily have also demonstrated similar pro and anti-tumor effects depending on origin of the tumor, stage of the tumor and the cellular context such as epigenetic status and transcriptional regulators (Eling et al., 2006; Matsumura et al., 2011). The molecular mechanism of GDF15 downstream signaling pathway remains to be resolved and this could potentially explain some of these pro and anti-tumorigenic roles. GDF15 also exists in several forms and both pro-domain and mature domain of GDF15 may have different biological activities (Bauskin et al., 2000, 2005). The expression of these different forms can vary at different stages of tumor progression potentially leading to contradicting functions. In addition, depending on the cellular localization, GDF15 might perform different functions leading to divergent roles in cancer. Further studies are required to delineate the exact molecular mechanism.

## GDF15 and Cardiovascular Systems

Several studies suggested GDF15 is protective in the heart. Xu et al. (2006) over-expressed Gdf15 through adenovirus in neonatal cardiomyocyte cultures and the cells became resistant to agonist-induced hypertrophy. Furthermore, they generated a cardiac-specific Gdf15 transgenic mouse line. Transgenic mice were otherwise normal but were protected from pressure overload hypertrophy. Conversely, Gdf15 deficient mice also appear normal, but they demonstrated significantly greater cardiac hypertrophy following pressure overload stimulation. In a separate study, Gdf15 deficient mice were subjected to transient coronary artery ligation for 1 h followed by reperfusion for 24 h. The size of the area-at-risk during coronary occlusion was comparable to the wild-type littermates, however, myocardial infarct sizes after reperfusion were significantly larger in the Gdf15 deficient mice (Kempf et al., 2006).

Besides the heart, the effect of GDF15 on atherosclerosis has also been evaluated by several groups, but these results are contradictory. In one study, after treating LDL receptor deficient mice with a lethal body radiation, the animals were transplanted with bone marrow from Gdf15 deficient mice or wild type controls and put on an atherogenic diet for 24 weeks. While no difference in lesion size was observed, LDL-receptor knockout mice transplanted with bone marrow from Gdf15 deficient mice showed enhanced macrophage accumulation and features of atherosclerotic plaque destabilization, suggesting a protective role of GDF15 in advanced atherosclerosis and macrophage accumulation (Preusch et al., 2013). However, a similar study came to the opposite conclusion. LDL-R KO mice transplanted with bone marrow from Gdf15 deficient mice resulted in a reduction of early atherosclerotic lesion size after 4 weeks on the high fat diet. Their plaques had reduced macrophage infiltrates and decreased necrotic core formation (de Jager et al., 2011).

Another supporting evidence for the protective role of GDF15 on atherosclerosis came from a study using ApoE deficient mouse model of atherosclerosis. Johnen et al. (2012) investigated the effect of transgenic overexpression of GDF15 in macrophages. After 6 months of high-fat diet, ApoE deficient mice overexpressing GDF15 had significantly smaller lesions in the aortic sinus and the thoracic aorta. At the site of the abdominal aorta, lesion size was also reduced. On the contrary, when Gdf15 deficient mice were bred with ApoE knockout mice, Bonaterra et al. (2012) found that a loss of GDF15 led to reduced development of atherosclerotic lesions in the aortic arch and the innominate artery after the animals were put on a cholesterol-enriched diet for 20 weeks, suggesting that GDF15 may stimulate atherosclerotic lesion progression.

In summary, despite evidence from epidemiological studies that show circulating GDF15 levels are strongly associated with worsening cardiovascular prognosis, the exact function of GDF15 on the cardiovascular systems is still not clearly understood.

## GDF15 and Kidney

Circulating GDF15 levels have been shown to strongly correlate with increased risk of chronic kidney disease (CKD) progression. In the study of two independent, well characterized CKD cohorts from Clinical Phenotyping and Resource Biobank (C-PROBE) study (224 participants, mean age 58) and the Seattle Kidney Study (SKS) (297 participants, mean age 62), Nair et al. (2017) tested whether kidney tissue expression of GDF15 mRNA correlates with circulating levels of GDF15 and the association between circulating GDF15 and kidney function. 24 patients were selected from the C-PROBE cohort with matching plasma samples and gene expression data derived from kidney biopsy samples. Tubulointerstitial GDF15 mRNA was strongly and positively correlated with circulating GDF15 protein. From both cohorts, circulating GDF15 associated with a 30% decline in eGFR or progression to end stage renal disease (ESRD). Participants with higher serum GDF15 had greater risk of longitudinal decline in eGFR.

Preclinical studies provided some evidence that GDF15 could be renal protective. Renal damage was assessed in GDF15 deficient mouse models of both type 1 and type 2 diabetes. In streptozotocin induced type 1 diabetes mice, loss of GDF15 led to increased urinary glucose loss, increased urine production, increased α-SMA staining, increased type 1 collagen mRNA expression, increased KIM-1 expression, and increased expression of inflammatory markers. In *db/db* type 2 diabetes mice, genetic deletion of GDF15 results in higher level of urinary glucose loss and serum creatinine levels. Immunohistological analysis also demonstrated increased interstitial α-SMA staining, and KIM-1 staining in tubules, suggesting diabetic GDF15 deficient mice have substantially more tubular damage than diabetic wild type mice (Mazagova et al., 2013). A more recent study investigated the effects of recombinant GDF15 protein on kidney fibrosis using a renal fibrosis mouse model. After unilateral ureter obstruction (UUO) surgery, mice were treated twice weekly through intraperitoneal injection of recombinant GDF15 protein. After 10 days, kidney tissues were excised and stained with Masson’s tri-chrome and Sirius to evaluate renal fibrosis. α-SMA and collagen 1 were also stained to evaluate fibroblast activation. The fibrotic burden and fibroblast activation were significantly reduced in the kidneys from GDF15 treated mice (Kim Y.I. et al., 2018).

Although epidemiological studies clearly demonstrated a clear association between high circulation GDF15 levels and increased risks in cardiovascular and kidney diseases, they do not necessary contradicts with the protective roles played by GDF15 in heart and kidney suggested by the functional studies in the animal models. Upregulation of GDF15 might be body’s compensatory response to the tissue injury. A similar example is natriuretic peptide, which induces anti-hypertrophic and blood-pressure lowering effects, and yet is a biomarker of adverse outcomes (Woods, 2004; van der Velde et al., 2014).

## GDF15 and Metabolism

Baek et al. (2006), first noted the impact of GDF15 on body weight when they examined its anti-tumorigenic effects in ubiquitously overexpressing GDF15 mice. Both male and female mice had significantly reduced body weight and fat mass, however, no impact on food intake was observed. In mice, over expression of GDF15 led to decreased food intake and increased energy expenditure (Macia et al., 2012; Chrysovergis et al., 2014) and this improved metabolic state led to decreased inflammatory cytokines and increased lifespan on both normal chow diet and a high fat diet (Wang X. et al., 2014). Conversely, GDF15 deficient mice were shown to have increased body weight, fat mass and food intake illustrating that endogenous GDF15 is a physiologically relevant regulator of metabolism (Tsai et al., 2013). This is further reinforced by Kim K.H. et al. (2018) who recently showed that GDF15 deficient mice are prone to increased deposition of lipid in liver and development of symptoms resembling nonalcoholic fatty liver disease (NASH/NAFLD) whereas GDF15 overexpressing transgenic mice were resistant.

The increase in GDF15 production in prostate tumors and the resulting increase in its’ circulating levels following tumor implantation in mice, led Johnen et al. (2007) to examine whether GDF15 could be responsible for cancer anorexia and weight loss. Mice with tumor implants that increased GDF15 into the range found normally circulating in humans were able to gain weight, however; those with increasingly higher levels of circulating GDF15 were found to lose weight progressively. Furthermore, administration of recombinant GDF15 recapitulated these effects and this weight loss could be blocked with an anti-GDF15 antibody showing that these effects were specifically mediated by the increase in circulating GDF15 levels (Johnen et al., 2007). The observation that a correlation exists in patients with prostate cancer between the magnitude of their weight loss and the levels of circulating GDF15 but not IL6 strongly suggested this relationship translates to humans (Johnen et al., 2007). These observations, initiated a considerable effort to develop therapeutics targeted to the GDF15 system including anti-GDF15 antibodies proposed as a novel therapeutic strategy to treat cancer – induced cachexia (Gyuris, Lerner, Lin, WO2016/049470), the effort to deorphanize the GDF15 receptor (discussed section “Conclusion”) and the development of novel GDF15 analogs for the treatment of obesity and diabetes (Xiong et al., 2017).

## GDF15 Receptor Discovery

The suggestion that GDF15 is an important regulator of body weight in humans (Johnen et al., 2007) unleashed a worldwide search for the receptor (s) underlying these effects. GDF15 was initially suggested to mediate its effect on food intake and body weight through interaction with the TGFβ receptor, TGFβR2 (Johnen et al., 2007). However, subsequent efforts both supported (Wang C.Y. et al., 2014; Artz et al., 2016) and were unable to confirm this finding (Unsicker et al., 2013; Hsu et al., 2017; Mullican et al., 2017; Yang et al., 2017). Unfortunately, a recent finding that commercial sources of GDF15 may be contaminated with a low level of TGFβ may have stifled progress in this endeavor (Olsen et al., 2017). Our inability to confirm the interaction with TGFBRII led us and others to search for additional partners for GDF15. Two strategies were utilized to simultaneously identify the GDF15 receptor by four research groups. Since several orphan members of the GDNF receptor family have been described (Airaksinen and Saarma, 2002), we undertook a systematic approach to screen ligands against these receptors. Screening of GDF15 against GDNF receptors and the orphan GDNF receptors GFRAL and GAS1 established a specific interaction with GFRAL (Emmerson et al., 2017). Additionally, using custom in-house and commercially available broad screening arrays of cell surface receptors three groups identified a single protein, GFRAL, as the receptor for GDF15 (Hsu et al., 2017; Mullican et al., 2017; Yang et al., 2017). GFRAL was originally identified as an orphan member of the GDNF family (Li et al., 2005). As a single transmembrane cell surface protein, GFRAL does not appear to signal on its own and requires interaction with the co-receptor RET similar to other members of the GDNF receptor family (Hsu et al., 2017). GDF15 mediated activation of RET phosphorylation induces signaling through ERK and AKT pathways (Hsu et al., 2017; Mullican et al., 2017; Yang et al., 2017). Yang et al. (2017) further suggested that GFRAL may interact differentially with isoforms of RET with its most efficient coupling to RET51. The metabolic effects of GDF15 were found by all four groups to be dependent on interaction with GFRAL as the anorexigenic effects of GDF15 were consistently lost in GFRAL deficient mice (Emmerson et al., 2017; Hsu et al., 2017; Mullican et al., 2017; Yang et al., 2017). To eliminate the possibility that GFRAL deficient mice were refractory to GDF15 for reasons other than a direct interaction, we also utilized an anti-GFRAL antagonist monoclonal antibody and illustrated that the *in vivo* effects of GDF15 were also dependent on binding to GFRAL (Emmerson et al., 2017). Hsu et al. (2017) demonstrated that GFRAL deficient mice are resistant to the effects of cisplatin further supporting the involvement of the GDF15/GFRAL pathway in the anorexigenic effects of chemotherapeutic agents. Interestingly, although GDF15 was found to be expressed broadly in many tissues, GFRAL expression was found to be restricted to the brain with highest expression in the brainstem area postrema (Emmerson et al., 2017; Hsu et al., 2017; Mullican et al., 2017; Yang et al., 2017). GDF15 administration was found to specifically activate GFRAL-RET positive neurons in the area postrema as measured by increased c-Fos expression (Hsu et al., 2017). Taken together these results further confirm previous work illustrating the importance of the area postrema as the primary point of interaction for GDF15 (Tsai et al., 2014; Borner et al., 2016). Additionally, this restricted expression was found to be conserved across many species including mouse, rat, monkey and human (Emmerson et al., 2017; Hsu et al., 2017; Mullican et al., 2017; Yang et al., 2017) suggesting that the effect of GDF15 on food intake and body weight is a phylogenetically conserved system for metabolic control.

## Conclusion

Significant progress has been made on GDF15, a remote member of TGFβ superfamily, ever since its discovery more than two decades ago. GDF15 is highly regulated under stressed condition and can be utilized as a biomarker for various diseases. As to its function, there are multiple studies connecting GDF15 with different roles in its target issues, including heart, kidney, brain and tumors. However, the results are sometimes not consistent or even contradictory. Recent identification of the endogenous receptor for GDF15 may allow investigators to shed more light on the mechanism of action of GDF15 in metabolism. However, more work is needed to further elucidate the underlying molecular mechanisms of GDF15 in other pathophysiological states.

## Author Contributions

All authors listed have made a substantial, direct and intellectual contribution to the work, and approved it for publication.

## Conflict of Interest Statement

All the authors are working at Eli Lilly and Company.
